# Chronic diseases in the geriatric population: morbidity and use of primary care services according to risk level

**DOI:** 10.1186/s12877-021-02217-7

**Published:** 2021-04-26

**Authors:** Jaime Barrio-Cortes, Almudena Castaño-Reguillo, María Teresa Beca-Martínez, Mariana Bandeira-de Oliveira, Carmen López-Rodríguez, María Ángeles Jaime-Sisó

**Affiliations:** 1 Research Unit, Primary Health Care Management of Madrid, Madrid, Spain; 2 Foundation for Biosanitary Research and Innovation in Primary Care, Madrid, Spain; 3 Faculty of Health, Camilo José Cela University, Madrid, Spain; 4 Healthcare Centre Ciudad Jardín, Primary Health Care Management of Madrid, Madrid, Spain; 5 Preventive Medicine Department, Virgen de la Salud Hospital, Toledo Hospital Complex, Toledo, Spain

**Keywords:** Geriatrics, Chronic disease, Use of services, Primary health care, Morbidity group, Stratification

## Abstract

**Background:**

Geriatric patients have significant morbidity and greater needs for care and assistance. The objective of this study was to describe the characteristics, morbidity, and use of services in primary care (PC) of patients with chronic diseases older than 65 years according to their risk level assigned by the adjusted morbidity groups (AMG) and to analyse the factors associated with the use of PC services.

**Methods:**

This was a cross-sectional descriptive observational study. Patients older than 65 years from a healthcare service area, classified as chronically ill by the AMG classification system of the PC electronic medical record of the Community of Madrid, were included. Sociodemographic, clinical-care, and PC service utilization variables were collected. Univariate, bivariate and multivariate analyses were done.

**Results:**

A total of 3292 chronic patients older than 65 years were identified, of whom 1628 (49.5%) were low risk, 1293 (39.3%) were medium risk and 371 (11.3%) were high risk. Their mean age was 78.1 (SD = 8.1) years and 2167 (65.8%) were women. Their mean number of chronic diseases was 3.8 (SD = 2), 89.4% had multimorbidity and 1550 (47.1%) were polymedicated. The mean number of contacts/year with PC was 19.5 (SD = 18.2) [men: 19.4 (SD = 19.8); women: 19.5 (SD = 17.4)]. The mean number of contacts/year in people over 85 years was 25.2 (SD = 19.6); in people 76–85 years old, it was 22.1 (SD = 20.3); and in people 66–75 years old, it was 14.5 (SD = 13.9). The factors associated with greater use of services were age (B coefficient [BC] = 0.3; 95%CI = 0.2–0.4), high risk level (BC = 1.9; 95%CI =0.4–3.2), weight of complexity (BC = 0.7; 95%CI = 0.5–0.8), and ≥ 4 chronic diseases (BC = 0.7; 95%CI = 0.3–1.1).

**Conclusions:**

In the geriatric population, we found a high number of patients with chronic diseases and there were three levels of risk by AMG with differences in characteristics, morbidity, and use of PC services. The greatest use of services was by patients with older age, high risk level, greater weight of complexity and ≥ 4 chronic diseases. Further research is needed to develop an intervention model more adapted to the reality of the geriatric population based on risk levels by AMG.

**Supplementary Information:**

The online version contains supplementary material available at 10.1186/s12877-021-02217-7.

## Background

The United Nations categorizes all those who are over 65 years old in developed countries as older adults [1]. In Spain, these people represent a significant percentage of the total population, amounting to almost 9.3 million people, over 1.5 million of whom are over 85 years of age [2].

There is no single and homogeneous definition of chronic disease, although many studies do seem to agree on a non-self-limiting nature, an association with persistent and recurrent health problems, anatomical and functional limitations or sequelae, and a long duration of the disease (months or years and not days or weeks) [3–5]. Approximately 42% of the Spanish population suffers from at least one chronic process, including 70% of those over 65, with a mean of four chronic diseases per person [6]. Multimorbidity also has different definitions, but all agree on the association of two or more concomitant chronic diseases in a patient [7–9]. The majority of the Spanish older adult population meets this criterion [10].

In recent decades, the global epidemiological pattern has progressively shifted to more chronic diseases instead of acute diseases, which has led to an increase in the use of social health services, both in primary care (PC) and in hospitals. This rise in health services utilization is attributed to follow-up and secondary prevention programs for chronic patients as well as emergency room visits, and hospitalizations for decompensation or acute worsening of chronic conditions [11]. Some studies estimate that patients with chronic diseases generate 80% of PC consultations, 60% of hospital admissions, 33% of emergency service visits, and up to 70% of healthcare spending [12].

A basic element of the strategies for treating chronic patients is the implementation of population morbidity classification systems based on predictive models from the PC electronic medical records. These tools stratify the population into different categories according to their morbidity and use of services, and their aim is to help plan the care model and identify, from the PC data, those individuals at higher risk so they can receive proactive and specific interventions. These interventions allow us to satisfy the social health needs of the most complex and older adults patients, improving the equity of health care and managing health resources more efficiently [13]. In Spain, morbidity classification systems such as the Clinical Risk Groups (CRG) [14] and the Adjusted Clinical Groups (ACG) have been used [15]. In recent years, the Adjusted Morbidity Groups (AMG) classification system [16] has been implemented in 13 autonomous communities as part of the strategies of care for patients with chronic diseases [17]. The AMG is an isocomplexity population classification case-mix tool that allows grouping of chronic patients according to their morbidity and complexity. In this way they are classified into mutually exclusive categories according to their level of risk following the Kaiser Permanente pyramid model (chronic patients with high risk, medium risk, low risk, and without relevant chronic pathology) [18]. According to these AMG risk level and with the knowledge of the patient and their context, the PC health professional responsible for the patient care determines the level of intervention to assign in the PC electronic clinical record and, therefore, the care plan to implement in order to offer a more personalized attention. Although there are studies on the usefulness of AMG and the stratification of chronic patients into different risk levels in the general population [16, 19, 20], there is little information on its specific use in the geriatric population [17, 21].

The main objective of this study was to describe the characteristics, morbidity, and use of services of chronic patients over 65 years of age in a healthcare service areaaccording to their risk level assigned by the AMG, as well as to analyse the factors associated with the use of PC services.

## Methods

This was a cross-sectional descriptive observational study with an analytical approach. Patients older than 65 years identified as chronic by the AMG in the Ciudad Jardin health centre located in the Chamartín district of Madrid were included. This health centre treated a total population of 18,107, of whom 3863 were older than 65 years as of June 30, 2015. This district had a total population of 143,424 people, a mean age of 45 years (23% over 65), 55% women, 8.9% foreigners, and a MEDEA deprivation index located in quartile 1, which corresponds to the neighbourhoods with the lowest degree of deprivation in Madrid [22].

The AMG is a population classification system incorporated into the PC electronic medical record of the Community of Madrid (“AP-Madrid”). It considers as chronic all patients of any age who present at least one of the chronic diseases described in Additional file 1 [23]. The AMG assignment is based on the results on morbidity group and the level of complexity. The morbidity group classifies the population into mutually exclusive groups based on the diagnostic codes recorded in AP-Madrid for each patient by the health professionals responsible for their care. The complexity is calculated by the analysis of different variables, such as risk of mortality, admissions, visits to PC, and prescriptions, that are linked to the patient’s diagnoses and assigns the patient a numerical value of their complexity (complexity index). This index, allows the population to be stratified into three risk levels (high, medium, and low risk) as well as the subset without relevant chronic pathology (assigning four cut-off points obtained from the 40th, 70th, 85th, and 95th percentiles of the entire population) [16–18]. Besides, the AMG offers the PC doctor a summary label of the pathologies.

The variables studied were: a) sociodemographic variables (age, sex, region of origin), b) clinical care variables (immobilized at home, institutionalized in residence, need for a primary caregiver, home support, and palliative care [24], level of risk according to AMG, number and type of chronic diseases, multimorbidity, patient complexity index [numerical value of patient complexity assigned by AMG which is a index measured based on morbidity] [16], polymedication [patients with a medication regimen that implies having been prescribed five or more drugs for their chronic conditions as baseline treatment]), c) use of PC services (number of annual contacts, type of contact [administrative, laboratory or health], form of contact [face-to-face, telephone, home], and occupation of the contact professional [nurse, family doctor, dentist]). These variables were extracted from the information recorded in AP-Madrid database. Sociodemographic and clinical-care variables were recorded as of June 30, 2015, and the PC service use period was June 30, 2015 to June 30, 2016.

The prevalence of chronic patients in the basic health area was calculated, as well as their distribution into the high, medium, and low risk levels of AMG. A descriptive analysis of each variable was performed with frequencies and percentages for the qualitative data and with the mean (standard deviation), median (interquartile range), and test of normality for the quantitative data. For the bivariate analysis, the χ^2^ test was used for comparing qualitative variables (or Fisher’s exact test when appropriate), the Mann-Whitney U test was used for dichotomous and quantitative variables and the Kruskal-Wallis test was used for polytomous and quantitative variables. The statistical results of multiple testings were adjusted with the Bonferroni method. To analyse the factors associated with the use of PC services, a linear regression model was built, whose dependent variable was the number of total contacts with the health system and whose independent variables were those that were significantly associated in the univariate analysis or had clinical relevance. In order to simplify the analysis, regarding AMG risk levels, we made a dichotomous variable by combining low and medium risk compared with high risk. The results were considered statistically significant if *p* < 0.05. Data analysis was carried out with the statistical software IBM SPSS Statistics version 25.

The study has the approval of the Drug Research Ethics Committee of the La Princesa University Hospital and a favorable report from the Local Research Commission of the PC Management of the Community of Madrid.

## Results

The prevalence of geriatric patients with at least one chronic disease in the basic health area was 3292 (85.2%), of whom 1628 (49.5%) were classified by the AMG as low risk, 1293 (39.3%) as medium risk, and 371 (11.2%) as high risk. Their mean age was 78.1 (SD = 8.1) years, and 2167 (65.8%) were women. With respect to nationality 2801 (85.1%) were Spanish, 96 (2.9%) European, and 395 (12%) from the rest of the world. Regarding the clinical-care variables, 286 (8.7%) were immobilized, and 154 (4.7%) were institutionalized in residence. A total of 218 patients (6.6%) had a primary caregiver at home, 77 (2.3%) needed home support, and 32 (1%) were receiving palliative care. The mean number of chronic diseases was 3.8 (SD = 2), 2493 (89.4%) had multimorbidity, and 1550 (47.1%) were polymedicated. These global characteristics are divided by sex and age group in Table 1 and by risk level in Table 2.

**Table 1:** Sociodemographic and clinical-care characteristics of chronic patients > 65 years, overall and by sex and age groups

Variables n (%)	Total 3292 (100)	Men 1125 (34.2)	Women 2167 (65.8)	p	66–75 years 1409 (42.8)	76–85 years 1239 (37.6)	> 85 years 644 (19.6)	p
**Sociodemographic variables**								
Age^a^	78.1 (8.1)	77 (7.5)	78.6 (8.2)	< 0.01	70.31 (2.8)	80.82 (2.8)	89.87 (3.5)	< 0.01
Origin: Spain	2801 (85.1)	963 (85.6)	1838 (84.8)		1198 (85.0)	1046 (84.4)	557 (86.5)	
Another European country	96 (2.9)	29 (2.6)	67 (3.1)	0.7	36 (2.6)	35 (2.8)	25 (3.9)	0.1
Rest of the world	395 (12)	133 (11.8)	262 (12.1)		175 (12.4)	158 (12.8)	62 (9.6)	
**Clinical-care variables**								
Immobilized	286 (8.7)	68 (6)	218 (10.1)	< 0.01	15 (1.1)	97 (7.8)	174 (27)	< 0.01
Institutionalized	154 (4.7)	35 (3.1)	119 (5.5)	< 0.01	6 (0.4)	48 (3.9)	100 (15.5)	< 0.01
Primary Caregiver	218 (6.6)	58 (5.2)	160 (7.4)	0.01	16 (1.1)	75 (6.1)	127 (19.7)	< 0.01
Need for home support	77 (2.3)	20 (1.8)	57 (2.6)	0.12	4 (0.3)	25 (2.0)	48 (7.5)	< 0.01
Palliative Care	32 (1)	17 (1.5)	15 (0.7)	0.02	6 (0.4)	15 (1.2)	11 (1.7)	0.01
Level of risk								
Low	1628 (49.5)	522 (46.4)	1106 (51.0)		885 (62.8)	516 (41.6)	227 (35.2)	
Medium	1293 (39.3)	438 (38.9)	855 (39.5)	<0.01	450 (31.9)	553 (44.6)	290 (45.0)	<0.01
High	371 (11.2)	165 (14.7)	206 (9.5)		74 (5.3)	170 (13.7)	127 (19.7)	
Complexity index^a^	11.2 (8.9)	12.2 (9.9)	10.7 (8.2)	< 0.01	8.8 (6.6)	12.5 (9.6)	14.1 (10.3)	< 0.01
Number of chronic diseases^a^	3.8 (2.1)	3.6 (2)	4 (2)	< 0.01	3.4 (1.8)	4.2 (2.1)	4.3 (2.2)	< 0.01
Multimorbidity	2943 (89.4)	975 (86.7)	1968 (90.8)	< 0.01	1204 (85.5)	1142 (92.2)	597 (92.7)	< 0.01
Polymedicated	1550 (47.1)	471 (41.9)	1079 (49.8)	< 0.01	122 (8.7)	923 (74.5)	505 (78.4)	< 0.01

^a^ $$ \overline{\boldsymbol{x}} $$ (SD)

**Table 2:** Sociodemographic and clinical-care characteristics of chronic patients > 65 years, overall and according to risk level by adjusted morbidity groups (AMG)

Variables n (%)	Low risk 1628 (49.5)	Medium risk 1293 (39.3)	High risk 371 (11.2)	P
Sociodemographic variables				
Age^a^	76.2 (7.9)	79.2 (7.7)	82.2 (7.4)	< 0.01
Men	522 (32.1)	438 (33.9)	165 (44.5)	< 0.01
Women	1106 (67.9)	855 (66.1)	206 (55.5)	
Origin: Spain	1331 (81.8)	1146 (88.6)	324 (87.3)	
Another European country	58 (3.6)	32 (2.5)	6 (1.6)	< 0.01
Rest of the world	239 (14.7)	115 (8.9)	41 (11.1)	
Clinical-care variables				
Immobilized	44 (2.7)	123 (9.5)	119 (32.1)	< 0.01
Institutionalized	63 (3.9)	51 (3.9)	40 (10.8)	< 0.01
Primary Caregiver	23 (1.4)	100 (7.7)	95 (25.6)	< 0.01
Need for home support	12 (0.7)	38 (2.9)	27 (7.3)	< 0.01
Palliative Care	2 (0.1)	6 (0.5)	24 (6.5)	< 0.01
Number of chronic diseases^a^	2.6 (1.2)	4.5 (1.5)	7 (2.3)	< 0.01
Multimorbidity	1302 (80)	1270 (98.2)	371 (100)	< 0.01
Complexity index^a^	5.6 (2)	12.7 (2.7)	30.4 (12)	< 0.01
Polymedicated	466 (28.6)	767 (59.3)	317 (85.4)	< 0.01

^a^ $$ \overline{\boldsymbol{x}} $$ (SD)

The most prevalent chronic pathologies within the study population were high blood pressure (67.6%), dyslipidaemia (58.8%), diabetes (21.5%), and osteoporosis (24.5%). These global characteristics are divided by sex and age group in Table 3 and by risk level in Table 4.

**Table 3:** Distribution of chronic comorbidities in chronic patients > 65 years of age during the year of study, overall and by sex and age groups

Chronic comorbidity n (%)	Total 3292 (100)	Men 1125 (34.2)	Women 2167 (65.8)	p	66–75 years 1409 (42.8)	76–85 years 1239 (37.6)	> 85 years 644 (19.6)	p
Anaemia	268 (8.1)	87 (7.7)	181 (8.4)	0.5	60 (4.3)	112 (9.0)	96 (14.9)	< 0.01
Human immunodeficiency virus (HIV)	4 (0.1)	4 (0.4)	0 (0.0)	< 0.01	4 (0.3)	0 (0.0)	0 (0.0)	0.07
Cirrhosis	210 (6.4)	73 (6.5)	137 (6.3)	0.8	111 (7.9)	72 (5.8)	27 (4.2)	< 0.01
Gastrointestinal ulcer	90 (2.7)	46 (4.1)	44 (2)	< 0.01	33 (2.3)	44 (3.6)	13 (2)	0.08
Inflammatory bowel disease	25 (7.6)	12 (1.1)	13 (0.3)	0.1	14 (1.0)	8 (0.6)	3 (0.5)	0.4
Aortic aneurysm	39 (1.2)	33 (2.9)	6 (0.3)	< 0.01	6 (0.4)	23 (1.9)	10 (1.6)	< 0.01
Ischaemic heart disease	285 (8.7)	171 (15.2)	114 (5.3)	< 0.01	83 (5.9)	126 (10.2)	76 (11.8)	< 0.01
Dysrhythmias	512 (15.6)	204 (18.1)	308 (14.2)	< 0.01	108 (7.7)	233 (18.8)	171 (26.6)	< 0.01
Hypertension	2224 (67.6)	789 (70.1)	1435 (66.2)	0.02	801 (56.8)	910 (73.4)	513 (79.7)	< 0.01
Stroke	204 (6.2)	88 (7.8)	116 (5.4)	< 0.01	44 (3.1)	95 (7.7)	65 (10.1)	< 0.01
Heart chronic failure	217 (6.6)	73 (6.5)	144 (6.6)	0.9	34 (2.4)	87 (7.0)	96 (14.9)	< 0.01
Valvular heart disease	144 (4.4)	52 (4.6)	92 (4.2)	0.6	47 (3.3)	52 (4.2)	45 (7.0)	< 0.01
Obstructive chronic pulmonary disease	268 (8.1)	148 (13.2)	120 (5.5)	< 0.01	95 (6.7)	119 (9.6)	54 (8.4)	0.03
Asthma	172 (5.2)	30 (2.7)	142 (6.6)	< 0.01	78 (5.5)	68 (5.5)	26 (4)	0.3
Arthritis	96 (2.8)	24 (2.1)	72 (3.3)	0.05	38 (2.7)	37 (3.0)	21 (3.3)	0.8
Osteoarthritis	707 (21.5)	157 (14.0)	550 (25.4)	< 0.01	284 (20.2)	294 (23.7)	129 (20.0)	0.05
Osteoporosis	806 (24.5)	28 (2.5)	778 (35.9)	< 0.01	361 (25.6)	308 (24.9)	137 (21.3)	0.1
Dementia	203 (6.2)	45 (4.0)	158 (7.3)	< 0.01	14 (1.0)	78 (6.3)	111 (17.2)	< 0.01
Epilepsy	57 (1.7)	18 (1.6)	39 (1.8)	0.7	15 (1.1)	28 (2.3)	14 (2.2)	0.04
Parkinson	75 (2.3)	34 (3.0)	41 (1.9)	0.04	21 (1.5)	38 (3.1)	16 (2.5)	0.02
Alcohol abuse	106 (3.2)	77 (6.8)	29 (1.3)	< 0.01	57 (4.0)	33 (2.7)	16 (2.5)	0.07
Substance abuse	5 (0.1)	2 (0.2)	3 (0.1)	0.8	2 (0.1)	3 (0.2)	0 (0.0)	0.4
Anxiety	607 (18.4)	128 (11.4)	479 (22.1)	< 0.01	296 (21.0)	217 (17.5)	94 (14.6)	< 0.01
Depression	525 (15.9)	104 (9.2)	421 (19.4)	< 0.01	206 (14.6)	201 (16.2)	118 (18.3)	0.1
Diabetes Mellitus	708 (21.5)	322 (28.6)	386 (17.8)	< 0.01	280 (19.9)	293 (23.6)	135 (21.0)	0.06
Dyslipidaemia	1937 (58.8)	652 (58.0)	1285 (59.3)	< 0.01	822 (58.3)	788 (63.6)	327 (50.8)	< 0.01
Obesity	683 (20.7)	224 (19.9)	459 (21.2)	0.4	294 (20.9)	287 (23.2)	102 (15.8)	< 0.01
Thyroid disorder	659 (20.0)	109 (9.7)	550 (25.4)	< 0.01	298 (21.1)	251 (20.3)	110 (17.1)	0.1
Renal chronic failure	128 (3.9)	60 (5.3)	68 (3.1)	< 0.01	15 (1.1)	62 (5.0)	51 (7.9)	< 0.01
Glaucoma	285 (8.7)	86 (7.6)	199 (9.2)	0.1	93 (6.6)	116 (9.4)	76 (11.8)	< 0.01
Active neoplasia	295 (8.9)	159 (14.1)	136 (6.3)	< 0.01	102 (7.2)	132 (10.7)	61 (9.5)	< 0.01
Breast neoplasia	37 (1.1)	1 (0.1)	36 (1.7)	< 0.01	12 (0.9)	17 (1.4)	8 (1.2)	0.4
Prostate neoplasia	55 (1.7)	55 (4.9)	0 (0.0)	< 0.01	20 (1.4)	23 (1.9)	12 (1.9)	0.6
Colorectal neoplasia	38 (1.2)	19 (1.7)	19 (0.9)	0.04	15 (1.1)	16 (1.3)	7 (1.1)	0.8
Skin neoplasia	37 (1.1)	13 (1.2)	24 (1.1)	0.9	10 (0.7)	20 (1.6)	7 (1.1)	0.9
Lung neoplasia	24 (0.7)	13 (1.2)	11 (0.5)	0.04	11 (0.8)	6 (0.5)	7 (1.1)	0.3
Lymphoma	32 (0.9)	10 (0.9)	22 (1.0)	0.7	10 (0.7)	19 (1.5)	3 (0.5)	0.03
Bladder neoplasia	23 (0.7)	21 (1.9)	2 (0.1)	< 0.01	9 (0.6)	8 (0.6)	6 (0.9)	0.7
Leukaemia	19 (0.5)	11 (1.0)	8 (0.4)	0.03	5 (0.4)	10 (0.8)	4 (0.6)	0.3

**Table 4:** Distribution of chronic comorbidities in chronic patients > 65 years during the year of study overall and according to risk level by adjusted morbidity groups (AMG)

Chronic comorbidity n (%)	Low risk 1628 (49.5)	Medium risk 1293 (39.3)	High risk 371 (11.2)	p
Anaemia	61 (3.7)	108 (8.4)	99 (26.7)	< 0.01
Human immunodeficiency virus (HIV)	3 (0.2)	1 (0.1)	0 (0.0)	0.5
Cirrhosis	52 (3.2)	124 (9.6)	34 (9.2)	< 0.01
Gastrointestinal ulcer	40 (2.5)	31 (2.4)	19 (5.1)	0.01
Inflammatory bowel disease	6 (0.4)	15 (1.2)	4 (1.1)	0.04
Aorta aneurysm	2 (0.1)	19 (1.5)	18 (4.9)	< 0.01
Ischaemic heart disease	43 (2.6)	140 (10.8)	102 (27.5)	< 0.01
Dysrhythmias	173 (46.6)	254 (19.6)	85 (5.2)	< 0.01
Hypertension	921 (56.6)	973 (75.3)	330 (88.9)	< 0.01
Stroke	29 (1.8)	88 (6.8)	87 (23.5)	< 0.01
Heart chronic failure	11 (0.7)	91 (7.0)	115 (31.0)	< 0.01
Valvular heart disease	20 (1.2)	51 (3.9)	73 (19.7)	< 0.01
Obstructive chronic pulmonary disease	47 (2.9)	130 (10.1)	91 (24.5)	< 0.01
Asthma	60 (3.7)	92 (7.1)	20 (5.4)	< 0.01
Arthritis	17 (1.0)	58 (4.5)	21 (5.7)	< 0.01
Osteoarthritis	247 (15.2)	362 (28.0)	98 (26.4)	< 0.01
Osteoporosis	347 (21.3)	365 (28.2)	94 (25.3)	< 0.01
Dementia	57 (3.5)	90 (7.0)	56 (15.1)	< 0.01
Epilepsy	17 (1.0)	24 (1.9)	16 (4.3)	< 0.01
Parkinson	16 (1.0)	43 (3.3)	16 (4.3)	< 0.01
Alcohol abuse	28 (1.7)	48 (3.7)	30 (8.1)	< 0.01
Substance abuse	0 (0.0)	1 (0.1)	4 (1.1)	< 0.01
Anxiety	260 (16.0)	278 (21.5)	69 (18.6)	< 0.01
Depression	163 (10.0)	271 (21.0)	91 (24.5)	< 0.01
Diabetes Mellitus	198 (12.2)	344 (26.6)	166 (44.7)	< 0.01
Dyslipidaemia	839 (51.5)	840 (65.0)	258 (69.5)	< 0.01
Obesity	241 (14.8)	333 (25.8)	109 (29.4)	< 0.01
Thyroid disorder	275 (16.9)	292 (22.6)	92 (24.8)	< 0.01
Renal chronic failure	5 (0.3)	32 (2.5)	91 (24.5)	< 0.01
Glaucoma	111 (6.8)	130 (10.1)	44 (11.9)	< 0.01
Active neoplasia	36 (2.2)	137 (10.6)	122 (32.9)	< 0.01
Breast neoplasia	4 (0.2)	15 (1.2)	18 (4.9)	< 0.01
Prostate neoplasia	7 (0.4)	27 (2.1)	21 (5.7)	< 0.01
Colorectal neoplasia	2 (0.1)	22 (1.7)	14 (3.8)	< 0.01
Skin neoplasia	7 (0.4)	19 (1.5)	11 (3)	< 0.01
Lung neoplasia	4 (0.1)	8 (0.6)	14 (3.8)	< 0.01
Lymphoma	7 (0.4)	13 (1.0)	12 (3.2)	< 0.01
Bladder neoplasia	1 (0.1)	8 (0.6)	14 (3.8)	< 0.01
Leukaemia	1 (0.1)	10 (0.8)	8 (2.2)	< 0.01

As for the use of PC services, the mean number of annual contacts in patients older than 65 years was 19.5 (SD = 18.2), with a mean of 25.2 (SD = 19.6) in those over 85 years of age, of 22.1 (SD = 20.3) in the age group of 76–85 years, and of 14.5 (SD = 13.9) in the 66–75 group. Regarding to the type of contact, a mean of 16.8 (SD = 6.5) was medical and 1.5 (SD = 4.4) was administrative.

According to the contact form, a mean of 17 (SD = 14.6) was face-to-face contacts. The mean number of visits to the family doctor was 9.7 (SD = 8.1), 6.5 (SD = 9.9) to the nurse, and 0.2 (SD = 0.9) to the social worker. These data on the use of global PC services are divided by sex and age group in Table 5 and by risk level in Table 6.

**Table 5:** Use of annual services by chronic patients > 65 years by sex and age groups

n (%)	Total 3292 (100)	Men 1125 (34.2)	Women 2167 (65.8)	p	66–75 years 1409 (42.8)	76–85 years 1239 (37.6)	> 85 years 644 (19.6)	p
**Total annual contacts with primary care** $\overline{x}$ (SD)	19.5 (18.2)	19.4 (19.8)	19.5 (17.4)	0.1	14.5 (13.9)	22.3 (20.3)	25.2 (19.6)	< 0.01
**Type of contact**								
Medical	16.8 (16.2)	16.6 (17.9)	16.9 (15.29)	0.03	12.7 (12.3)	19.2 (18.4)	21.3 (17.2)	< 0.01
Administrative	1.5 (4.5)	1.8 (5)	1.4 (4.2)	0.4	0.9 (3.3)	1.8 (4.8)	2.5 (5.7)	< 0.01
Laboratory	1.1 (1.6)	1 (1.5)	1.2 (1.7)	0.04	1 (1.3)	1.2 (1.8)	1.3 (2)	0.01
**Form of contact**								
Face-to-face	17 (14.6)	17.3 (15.8)	16.9 (13.9)	0.7	13.7 (12.6)	19.7 (16)	19.1 (14.4)	< 0.01
Telephone	0.8 (3.5)	0.6 (2.3)	0.9 (4)	< 0.01	0.3 (2)	0.9 (4.3)	1.7 (4.1)	< 0.01
Home	1.7 (6.5)	1.6 (7.9)	1.7 (5.5)	< 0.01	0.4 (3.1)	0.9 (4.3)	1.7 (4.1)	< 0.01
**Professional contacted**								
Family physician	9.7 (8.1)	9.2 (7.2)	9.9 (8)	0.04	7.6 (6.5)	10.9 (8.7)	11.8 (8.9)	< 0.01
Nurse	6.5 (9.9)	6.8 (12.6)	6.3 (8.2)	0.6	4.3 (6.5)	7.6 (12.3)	8.9 (10)	< 0.01
Dentist	0.03 (0.3)	0.03 (0.3)	0.03 (0.3)	0.8	0.04 (0.3)	0.03 (0.3)	0.01 (0.1)	0.05
Physiotherapist	0.4 (2.5)	0.3 (2)	0.5 (2.7)	0.2	0.6 (2.8)	0.4 (2.4)	0.2 (2)	< 0.01
Social worker	0.2 (0.9)	0.1 (0.9)	0.2 (0.9)	0.1	0.1 (0.6)	0.2 (1.1)	0.3 (1.3)	< 0.01

**Table 6:** Use of annual services by chronic patients > 65 years according to risk level by adjusted morbidity groups (AMG)

n (%)	Low risk 1628 (49.5%)	Medium risk 1293 (39.3%)	High risk 371 (11.3%)	P
**Total annual contacts with primary care** $\overline{x}$ (SD)	13.1 (12.3)	23 (17.3)	35.3 (27.9)	< 0.01
**Type of contact**				
Medical	11.2 (10.7)	20 (15.3)	30.2 (25.7)	< 0.01
Administrative	0.9 (3.4)	1.8 (4.8)	3.3 (6.6)	< 0.01
Laboratory	0.9 (3.4)	1.2 (1.6)	1.9 (2.5)	< 0.01
**Form of Contact**				
Face-to-face	12.2 (11)	20.2 (14.7)	27 (19.4)	< 0.01
Telephone	0.3 (1.3)	1 (2.8)	2.6 (8.3)	< 0.01
Home	0.6 (3.1)	1.9 (5.7)	5.9 (13.9)	< 0.01
**Professional contacted**				
Family physician	6.7 (5.4)	11.5 (7.8)	16.4 (12.2)	< 0.01
Nurse	4 (6.5)	7.6 (9.1)	13.4 (17.9)	< 0.01
Dentist	0.02 (0.3)	0.05 (0.4)	0 (0)	0.01
Physiotherapist	0.4 (2.5)	0.6 (2.8)	0 (0)	< 0.01
Social worker	0.1 (0.6)	0.2 (1)	0.4 (1.6)	< 0.01

In the multivariate analysis, factors associated with the use of PC services in geriatric patients in the model was age (B coefficient [BC] = 0.3; 95% CI = 0.2–0.4), high risk level (BC = 1.9; 95% CI = 0.5–3.4), ≥ 4 chronic diseases (BC = 0.7; 95% CI = 0.3–1.2) and complexity index (BC = 0.7; 95% CI = 0.6–0.8) (Table 7).

**Table 7:** Factors associated with the use of services by patients > 65 years

Use of primary care services				
Variables	B Coefficient	95% CI		P
		Lower	Upper	
Age^a^	0.3	0.2	0.4	< 0.01
High risk level^b^	1.9	0.5	3.4	< 0.01
Complexity Index^a^	0.7	0.6	0.8	< 0.01
≥ 4 chronic diseases^b^	0.7	0.3	1.2	< 0.01

Linear regression R^2^ = 0.22

^a^Continuous variable

^b^Dichotomous variable

## Discussion

Some 85.2% of people over 65 years of age in the basic health area had chronic disease, figures similar to those of the National Health Survey of 2017 in Spain, which observed that 89.5% of people aged 65–74 years, 95.2% of those aged 75–84 years, and 96.5% of those aged over 85 years reported some disease or chronic health problem [25]. In other European countries, such as Italy, the figures also resemble those of our study, with 86% of those over 65 years of age having some chronic disorder [26]. These figures are higher than those reported by the National Center for Chronic Disease Prevention and Health Promotion of the United States, where 67.7% of those over 65 years belonging to the Medicare programme had at least one chronic disease [27]. The prevalence of chronic diseases was slightly higher than that observed in other studies that have used the AMG in Spain, such as that of the Chronicity Strategy Evaluation Report, where 77.6% of the population older than 65 years had at least one chronic health problem, a percentage that exceeded 80% in people older than 85 years [17].

This population of chronic patients older than 65 years had a high mean age; almost two-thirds (65.8%) were women; 89.4% had multimorbidity; and almost half (47.1%) were polymedicated. The predominance of women coincided with other series that showed a higher frequency of chronic diseases in women [28–30].

Women older than 65 years had greater immobility, more chronic diseases, and polypharmacy more often than men, as observed by other authors [31, 32]. Anxiety, depression, thyroid disorder, osteoporosis, obesity, high blood pressure, and dyslipidaemia predominated in women, while chronic obstructive pulmonary disease (COPD) and ischaemic heart disease predominated in men, in line with other studies [33, 34].

The mean age of our series was similar to that of other series of chronic patients older than 65 (74.9 years) [28, 35, 36]. Medium and high risk were more frequent in the age group older than 85 years (45 and 19.7%, respectively). High-risk patients presented a higher percentage of functional impairment and immobility than medium and low-risk patients, which has also been observed in complex chronic patients [31, 35, 37]. More high-risk chronic patients required primary caregivers than medium and low-risk chronic patients, but fewer than 40% of patients that had primary caregivers in other pluripathological series [31]. One series has reported a 0.4% rate of palliative care among chronically ill patients [38], lower to our data, and it was much more common in the high-risk stratum (6.5%). The mean number of chronic diseases was 2.6 in low-risk, 4.5 in middle-risk, and 7 in high-risk patients. The number of chronic diseases adjusted for age increased with age, as in other series [33, 39, 40]. The most frequent chronic diseases were similar to those in other series, with a cardiovascular, lung, neurological, kidney and osteoarticular disease predominance [34, 41, 42], and they were more severe in high and medium-risk chronic patients. Polypharmacy was high in high-risk patients (85.4% of patients), which is in line with the 50–94% of other studies [31, 41, 43] and is much higher than that observed in medium and low-risk patients (59.3 and 28.6%, respectively).

It’s remarkable that, while older adults from 65 to 75 years had only 9% of polymedication, older adults from 76 to 85 years experienced a high increase up to 75%. These can be explained because the potential negative health impacts of multimorbidity such as reduced quality of life and functional capacity, inadequate use of healthcare services and increased complications can be more pronounced in patients older than 75 years, needing a higher prescription of drugs in order to control their chronic diseases [44].

Thus, low-risk chronic geriatric patients have more often a single chronic disease, and medium-risk chronic patients normally are multipathological or multimorbid patients, terms that appear in the literature and that share frequencies and similar characteristics [31, 41]. High-risk chronic patients could be more specifically multipathological patients with functional and fragile deterioration or complex chronic patients, with a reported prevalence of these patients of 1.38–5% [45], similar to our finding.

The contact with PC of these chronic patients older than 65 years was high, similar to that observed in other studies with the same age [46] and higher than adults and children in other studies with AMG [11, 21]. The mean use of PC health services for these chronic patients was high at all risk levels and was much higher at high risk levels. The majority of contacts were health related and face-to-face, similar to the trend in other studies [11, 46–48]. The mean number of contacts with the family physician found in our study was lower than the mean number of medical consultations in other studies [49]. It was higher than the mean number of contacts with nursing, unlike other previous studies where the mean number of contacts with nursing was higher than the mean number of contacts with doctors [50]. This lower number of contacts with nursing by chronic patients should make us reflect on how care is being given to these patients, since the models of care that are proposed in the strategies for addressing chronicity prioritize care focussed on nursing and directing the intervention of the physician to processes that require medical care or situations of greater complexity [23]. In the same sense, visits to social workers were very rare, but it would be expected that the social care needs of some chronic patients would be higher, although the area covered by the centre overall is an area with good socioeconomic indicators. The availability of only one social worker for several centres can also influence the accessibility to the service. Also noteworthy is the low number of noncontact services and home visits, something that should be promoted in the framework of care for chronic geriatric patients as well as in the COVID-19 pandemic situation [51].

The two sexes had no significant differences in contact with PC despite other studies where was reported greater use of health-care services in women [32]. However, the different age groups did, annual contacts being higher in patients over 85 years of age than others, as is also observed in other studies [8, 11].

The increase in the use of PC services was significantly associated in the multivariate model with older age, high risk level, greater weight of complexity, and a number of chronic diseases ≥4, a profile similar to that of the complex chronic patient [31]. The variable which had a greatest impact on the PC services use was the high risk level. Thus, the AMG high risk level also help to identify patients with more need of care as happened in adult patients too and unlike pediatric patients where high risk level was not associated with more utilization of PC services [11, 20, 21].

As age influences in the PC services utilization, maybe older age could be taken more specifically in consideration by in the PC electronic clinical record when assigning the patient intervention level in the PC electronic clinical record along with the risk level by AMG.

Regarding the limitations, on the one hand, the basic health area was a neighbourhood with a medium–high socioeconomic level (MEDEA deprivation index in quartile 1) [22], which can make the population more likely to have double insurance that would reduce the use of health services of the public health system. However, more than 90% of people living in Madrid had access to public PC in 2015 [34]. The PC diagnostic information provides a good approximation of the morbidity of a population (especially chronic diseases), but it is not 100% complete, and some underreporting should be recognized since the coding of the diagnoses can vary according to the doctor who performs it. Despite all this, we have worked with real-world data, which provided a large volume of information from the population in real clinical practice conditions, overcoming the limitations of studies conducted with surveys or small samples. On the other hand, there are authors who have raised doubts about the transparency and complexity of AMG [52, 53], which has generated a debate on whether this situation is common to the rest of the commercial grouping tools [19, 54]. In addition, AMG has a clinical-healthcare management utility that considers the complexity and morbidity of the patient but does not take into account other factors, such as psychosocial problems. Even so, it has been a useful tool for measuring the burden of disease in PC, and the Ministry of Health aims to apply it to the entire National Health System for the management of chronic patients [16, 19].

In conclusion, almost 90% of the population older than 65 years from the health centre was classified by AMG as chronic. These chronic geriatric patients were stratified by AMG into three risk levels, which presented differences in sex, age, functional impairment, need for care, morbidity, complexity, polypharmacy, and use of PC services. The greatest use of services was by patients with older age, high risk level, greater weight of complexity, and ≥ 4 chronic diseases. Further research is needed to be able to develop an intervention model more adapted to the reality of the geriatric population based on risk levels by AMG.

## Supplementary Information

**Additional file 1.** Types of chronic diseases considered by the Adjusted Morbidity Group (AMG) in the Community of Madrid at the time of data extraction.

## Data Availability

The datasets generated and analysed during the current study are not publicly available due the belong to the Madrid Primary Care Electronic Clinical Record (AP Madrid) but are available from the corresponding author on reasonable request.

## References

[1] World Health Organization (2020). WHO Study on global AGEing and adult health (SAGE).

[2] Instituto Nacional de Estadística (2020). Proporción de personas mayores de cierta edad. Indicadores de Estructura de la Población.

[3] O’Halloran J, Miller GC, Britt H (2004). Defining chronic conditions for primary care with ICPC-2. Fam Pract.

[4] Goodman RA, Posner SF, Huang ES, Parekh AK, Koh HK (2013). Defining and Measuring Chronic Conditions: Imperatives for Research, Policy, Program, and Practice. Prev Chronic Dis.

[5] Valderas JM, Starfield B, Sibbald B, Salisbury C, Roland M (2009). Defining comorbidity: implications for understanding health and health services. Ann Fam Med.

[6] Sociedad Española de Medicina de Familia y Comunitaria (semFYC) (2018). Sociedad Española de Medicina Interna - SEMI. X Congreso Nacional de Atención Sanitaria al Paciente Crónico.

[7] Le Reste JY, Nabbe P, Manceau B, Lygidakis C, Doerr C, Lingner H (2013). The European general practice research network presents a comprehensive definition of multimorbidity in family medicine and long term care, following a systematic review of relevant literature. J Am Med Dir Assoc.

[8] Huntley AL, Johnson R, Purdy S, Valderas JM, Salisbury C (2012). Measures of multimorbidity and morbidity burden for use in primary care and community settings: a systematic review and guide. Ann Fam Med.

[9] Bernabeu-Wittel M, Alonso-Coello P, Rico-Blázquez M, Rotaeche del Campo R, Sánchez Gómez S, Casariego Vales E (2014). Desarrollo de guías de práctica clínica en pacientes con comorbilidad y pluripatología. Atención Primaria..

[10] Islam MM, Valderas JM, Yen L, Dawda P, Jowsey T, McRae IS (2014). Multimorbidity and comorbidity of chronic diseases among the senior Australians: prevalence and patterns. PLoS One.

[11] Barrio Cortes J, Suárez Fernández C, Bandeira de Oliveira M, Beca Martínez MT, Lozano Hernández C, Del Cura-González I (2019). Utilización de los servicios de salud de Atención Primaria en los pacientes crónicos según nivel de riesgo. Rev Esp Salud Publica..

[12] Empantanados Bengoa R (2008). Revista de Innovación Sanitaria y Atención Integrada.

[13] Contel JC, Muntané B, Camp L (2012). La atención al paciente crónico en situación de complejidad: El reto de construir un escenario de atención integrada. Aten Primaria.

[14] Estupiñán-Ramírez M, Tristancho-Ajamil R, Company-Sancho MC, Sánchez-Janáriz H (2019). Comparación de modelos predictivos para la selección de pacientes de alta complejidad. Gac Sanit.

[15] Orueta J-F, Urraca J, Berraondo I, Darpón J, Aurrekoetxea J-J (2006). Adjusted Clinical Groups (ACGs) explain the utilization of primary care in Spain based on information registered in the medical records: A cross-sectional study. Health Policy (New York).

[16] Monterde D, Vela E, Clèries M (2016). Los grupos de morbilidad ajustados: nuevo agrupador de morbilidad poblacional de utilidad en el ámbito de la atención primaria. Atención Primaria..

[17] Grupo de trabajo Ministerio de Sanidad Servicios Sociales e Igualdad (2018). Informe del proyecto de estratificación de la población por grupos de morbilidad ajustados (GMA) en el Sistema Nacional de Salud (2014-2016).

[18] González González AI, Miquel Gómez AM, Rodríguez Morales D, Hernández Pascual M, Sánchez Perruca L, Mediavilla HI (2017). Concordancia y utilidad de un sistema de estratificación para la toma de decisiones clínicas. Atención Primaria..

[19] Monterde D, Vela E, Clèries M, García Eroles L, Pérez SP (2019). Validez de los grupos de morbilidad ajustados respecto a los clinical risk groups en el ámbito de la atención primaria. Atención Primaria..

[20] Barrio-Cortes J, del Cura-González I, Martínez-Martín M, López-Rodríguez C, Jaime-Sisó MÁ, Suárez-Fernández C (2020). Grupos de morbilidad ajustados: características y comorbilidades de los pacientes crónicos según nivel de riesgo en Atención Primaria. Atención Primaria..

[21] Barrio Cortes J, Suárez Fernández C, Bandeira de Oliveira M, Muñoz Lagos C, Beca Martínez MT, Lozano Hernández C (2020). Enfermedades crónicas en población pediátrica: comorbilidades y uso de servicios en atención primaria. An Pediatría.

[22] Álvarez-del Arco D, Vicente Sánchez M, Alejos B, Pascual C, Regidor E (2013). Construcción de un índice de privación para los barrios de Madrid y Barcelona. Rev Esp Salud Publica.

[23] Servicio Madrileño de Salud. Estrategia de Atención a Pacientes con Enfermedades Crónicas en la Comunidad de Madrid. Madrid; 2013.

[24] Servicio Madrileño de Salud (2014). Cartera de servicios estandarizados de Atención Primaria de Madrid.

[25] Ministerio de Sanidad, Consumo y Bienestar social. Encuesta Nacional de Salud (ENSE) 2017. Madrid: Ministerio de Sanidad, Consumo y Bienestar social; 2018.

[26] Marengoni A, Bonometti F, Nobili A, Tettamanti M, Salerno F, Corrao S, Iorio A, Marcucci M, Mannucci PM, Italian Society of Internal Medicine (SIMI) Investigators (2010). In-hospital death and adverse clinical events in elderly patients according to disease clustering: the REPOSI study. Rejuvenation Res.

[27] Centers for Disease Control and Prevention (2018). National Center for Chronic Disease Prevention and Health Promotion.

[28] García-Olmos L, Salvador CH, Alberquilla Á, Lora D, Carmona M, García-Sagredo P (2012). Comorbidity patterns in patients with chronic diseases in general practice. PLoS One.

[29] Van Oostrom SH, Picavet HSJ, van Gelder BM, Lemmens LC, Hoeymans N, van Dijk CE (2012). Multimorbidity and comorbidity in the Dutch population - data from general practices. BMC Public Health.

[30] Van Minh H, Nawi N, Juvekar S, Razzaque A, Ashraf A, Hadi A (2008). Self-reported prevalence of chronic diseases and their relation to selected sociodemographic variables: a study in INDEPTH Asian sites, 2005. Prev Chronic Dis.

[31] Ramírez-Duque N, Ollero-Baturone M, Bernabeu-Wittel M, Rincón-Gómez M, Ortiz-Camuñez MÁ, García-Morillo S (2008). Características clínicas, funcionales, mentales y sociales de pacientes pluripatológicos. Estudio prospectivo durante un año en Atención Primaria. Rev Clínica Española.

[32] Redondo-Sendino Á, Guallar-Castillón P, Banegas JR, Rodríguez-Artalejo F (2006). Gender differences in the utilization of health-care services among the older adult population of Spain. BMC Public Health.

[33] Rocca WA, Boyd CM, Grossardt BR, Bobo WV, Finney Rutten LJ, Roger VL, Ebbert JO, Therneau TM, Yawn BP, St. Sauver JL (2014). Prevalence of multimorbidity in a geographically defined American population. Mayo Clin Proc.

[34] Esteban-Vasallo M, Dominguez-Berjon M, Astray-Mochales J, Genova-Maleras R, Perez-Sania A, Sanchez-Perruca L, Aguilera-Guzman M, Gonzalez-Sanz F (2009). Epidemiological usefulness of population-based electronic clinical records in primary care: estimation of the prevalence of chronic diseases. Fam Pract.

[35] Garin N, Olaya B, Perales J, Moneta MV, Miret M, Ayuso-Mateos JL, Haro JM (2014). Multimorbidity patterns in a national representative sample of the Spanish adult population. PLoS One.

[36] Rizza A, Kaplan V, Senn O, Rosemann T, Bhend H, Tandjung R (2012). Age- and gender-related prevalence of multimorbidity in primary care: the swiss fire project. BMC Fam Pract.

[37] Marengoni A, Angleman S, Melis R, Mangialasche F, Karp A, Garmen A, Meinow B, Fratiglioni L (2011). Aging with multimorbidity: a systematic review of the literature. Ageing Res Rev.

[38] Vega T, Arrieta E, Lozano JE, Miralles M, Anes Y, Gomez C, Quiñones C, Perucha M, Margolles M, Gómez de Caso JÁ, Gil M, Fernández S, de la Iglesia P, López A, Alamo R, Zurriaga O, Mauro Ramos J, por el Grupo RECENT (2011). Atención sanitaria paliativa y de soporte de los equipos de atención primaria en el domicilio. Gac Sanit.

[39] Sinnige J, Braspenning J, Schellevis F, Stirbu-Wagner I, Westert G, Korevaar J (2013). The prevalence of disease clusters in older adults with multiple chronic diseases – a systematic literature review. PLoS One.

[40] Barnett K, Mercer SW, Norbury M, Watt G, Wyke S, Guthrie B (2012). Epidemiology of multimorbidity and implications for health care, research, and medical education: a cross-sectional study. Lancet..

[41] Bernabeu-Wittel M, Barón-Franco B, Murcia-Zaragoza J, Fuertes-Martín A, Ramos-Cantos C, Fernández-Moyano A, Galindo FJ, Ollero-Baturone M (2011). A multi-institutional, hospital-based assessment of clinical, functional, sociofamilial and health-care characteristics of polypathological patients (PP). Arch Gerontol Geriatr.

[42] Violan C, Foguet-Boreu Q, Flores-Mateo G, Salisbury C, Blom J, Freitag M (2014). Prevalence, Determinants and Patterns of Multimorbidity in Primary Care: A Systematic Review of Observational Studies. PLoS One.

[43] Doos L, Roberts EO, Corp N, Kadam UT (2014). Multi-drug therapy in chronic condition multimorbidity: a systematic review. Fam Pract.

[44] Prados-Torres A, del Cura-González I, Prados-Torres JD, Muth C, Leiva-Fernández F, Lopez-Rodriguez JA (2020). MULTIPAP Study: Improving healthcare for patients with multimorbidity. Br J Gen Pract.

[45] Garin N, Olaya B, Moneta MV, Miret M, Lobo A, Ayuso-Mateos JL, Haro JM (2014). Impact of multimorbidity on disability and quality of life in the Spanish older population. PLoS One.

[46] Glynn LG, Valderas JM, Healy P, Burke E, Newell J, Gillespie P, Murphy AW (2011). The prevalence of multimorbidity in primary care and its effect on health care utilization and cost. Fam Pract.

[47] Salisbury C, Johnson L, Purdy S, Valderas JM, Montgomery AA (2011). Epidemiology and impact of multimorbidity in primary care: a retrospective cohort study. Br J Gen Pract.

[48] Vedsted P, Olesen F (2005). Social environment and frequent attendance in Danish general practice. Br J Gen Pract.

[49] Martín-Fernández J, Gómez-Gascón T, del Cura-González MI, Tomás-García N, Vargas-Machuca C, Rodríguez-Martínez G (2010). La calidad de vida relacionada con la salud como factor explicativo de la utilización de la consulta de medicina de familia: un estudio bajo el modelo conductual. Rev Esp Salud Publica..

[50] Martín-Fernández J, Rodríguez-Martínez G, Ariza-Cardiel G, Vergel Gutierrez MÁ, Hidalgo Escudero AV, Conde-López JF (2013). Variables que condicionan la utilización de la consulta de enfermería en centros de salud de la Comunidad de Madrid. Rev Esp Salud Publica..

[51] Martínez-Riera JR, Gras-Nieto E. Home Care and COVID-19. Before, in and after the state of alarm. Enferm Clin. 2021;31:S24–8. 10.1016/j.enfcli.2020.05.003. 10.1016/j.enfcli.2020.05.003 PMC7225710 32419772

[52] Inoriza JM, Carreras M, Pérez-Berruezo X, Coderch J (2017). Los grupos de morbilidad ajustados: un debate pendiente. Atención Primaria..

[53] Inoriza JM, Sánchez-Pérez I, Carreras M, Coderch J (2017). ¿Son los grupos de morbilidad ajustados concordantes con el criterio clínico de intervención en una estrategia de crónicos?. Atención Primaria..

[54] Monterde D, Vela E, Clèries M (2017). Respuesta a la carta «Los grupos de morbilidad ajustados: un debate pendiente». Atención Primaria.

