# Supplementary material for: Chronic diseases in the geriatric population: morbidity and use of primary care services according to risk level
Source: BMC Geriatr. 2021 Apr 26;21:278. doi: 10.1186/s12877-021-02217-7 (PMC8074273; doi:10.1186/s12877-021-02217-7)
Supplement: Supplementary file 1 — Additional file 1. Types of chronic diseases considered by the Adjusted Morbidity Group (AMG) in the Community of Madrid at the time of data extraction. [file 12877_2021_2217_MOESM1_ESM.docx]

**TITLE PAGE**

**Title:** Chronic diseases in the geriatric population: morbidity and use of primary care services according to risk level.

**Authors:**

Jaime Barrio Cortes (1,2,3) *

Almudena Castaño Reguillo (4)

María Teresa Beca Martínez (5)

Mariana Bandeira de Oliveira (4)

Carmen López Rodríguez (4)

María Ángeles Jaime Sisó (4)

(1) Primary Care Investigation Unit. Gerencia Asistencial de Atención Primaria. Madrid. Spain.

(2) Foundation for Biosanitary Research and Innovation in Primary Care. Madrid. Spain.

(3) Faculty of Health. Universidad Camilo José Cela. Madrid. España.

(4) Healthcare Centre Ciudad Jardín. Gerencia Asistencial de Atención Primaria. Madrid. Spain.

(5) Preventive Medicine Department. Hospital Virgen de la Salud. Complejo Hospitalario de Toledo. Spain.

***Corresponding Author:**

Jaime Barrio Cortes. MD, PhD. Primary Care Investigation Unit. Calle San Martín de Porres, 6. 5^th^ floor. 28035. Madrid, Spain.

Email: [jaime.barrio@salud.madrid.org](mailto:jaime.barrio@salud.madrid.org). Phone: (+34) 660117699.

**Appendix 1. Types of chronic diseases considered by the Adjusted Morbidity Group (AMG) in the Community of Madrid at the time of data extraction**

| Alcoholism |
| --- |
| Anaemia |
| Aorta aneurysm |
| Anxiety |
| Arthritis |
| Arthrosis |
| Asthma |
| Attention-Deficit/Hyperactivity Disorder (ADHD) |
| Bladder cancer |
| Breast cancer |
| Cardiopulmonary disease |
| Central nervous system cancer |
| Cervical cancer |
| Cirrhosis |
| Colon cancer |
| Dementia |
| Depression |
| Diabetes Mellitus |
| Dyslipidaemia |
| Dysrhythmias |
| Ear, nose and throat cancer |
| Endometrial cancer |
| Epilepsy |
| Gastrointestinal ulcer |
| Glaucoma |
| Heart chronic failure |
| Hepatoblastoma |
| Hodgkin/Other lymphomas |
| Human immunodeficiency virus (HIV) |
| Hyperlipidemia |
| Hypertension |
| Ischemic heart disease |
| Leukemia |
| Liver cancer |
| Lung cancer |
| Mental retardation |
| Multiple sclerosis |
| Obesity |
| Obstructive chronic pulmonary disease (OCPD) |
| Osteoarthritis |
| Osteoporosis |
| Pancreatic cancer |
| Parkinson |
| Prostate cancer |
| Renal cancer |
| Renal chronic failure |
| Retinoblastoma |
| Schizophrenia |
| Skin cancer |
| Soft tissues cancer |
| Stomach cancer |
| Stroke |
| Substance abuse |
| Testicle cancer |
| Thyroid cancer |
| Thyroid disorder |
| Ulcerative colitis |
| Valvular heart disease |
| Vasculitis |
